# STHLM3 NorDCaP: prospective multicentre trial of Stockholm3 for active surveillance biopsy guidance

**DOI:** 10.1111/bju.70366

**Published:** 2026-07-01

**Authors:** Nicola Giudici, Simone Scuderi, Thorgerdur Palsdottir, Tobias Nordstrom, Martin Eklund, Eva Ferlev Jensby, Michael Borre, Bodil Ginnerup Pedersen, Henrik Grönberg, Karina Dalsgaard Sørensen, Svein R Kjosavik, Anna Lantz

**Affiliations:** ^1^ Department of Molecular Medicine and Surgery Karolinska Institutet Stockholm Sweden; ^2^ Department of Pelvic Cancer Karolinska University Hospital Stockholm Sweden; ^3^ Department of Medical Epidemiology and Biostatistics Karolinska Institutet Stockholm Sweden; ^4^ Department of Clinical Sciences at Danderyd Hospital Karolinska Institutet Stockholm Sweden; ^5^ Department of Urology University Hospital Bern Bern Switzerland; ^6^ Unit of Urology/Division of Oncology, Gianfranco Soldera Prostate Cancer Lab IRCCS San Raffaele Scientific Institute Milan Italy; ^7^ Vita‐Salute San Raffaele University Milan Italy; ^8^ Department of Molecular Medicine Aarhus University Hospital Aarhus Denmark; ^9^ Department of Clinical Medicine Aarhus University Aarhus Denmark; ^10^ Department of Urology Aarhus University Hospital Aarhus Denmark; ^11^ Department of Radiology Aarhus University Hospital Aarhus Denmark; ^12^ The General Practice and Care Coordination Research Group Stavanger University Hospital Stavanger Norway

**Keywords:** Stockholm3, active surveillance, prostate cancer, risk stratification, surveillance biopsies

## Abstract

**Objectives:**

Active surveillance (AS) is the preferred approach for low‐risk prostate cancer (PCa). Recent efforts have explored new biomarkers to reduce the frequency of surveillance biopsy. This study assessed Stockholm3 for predicting PCa upgrading during surveillance biopsies.

**Materials and Methods:**

The prospective STHLM3‐AS NorDCaP trial included 199 men with International Society of Urological Pathologists grade group 1 (ISUP GG 1) PCa under AS in Sweden, Denmark, and Norway (2019–2022). All men had ISUP GG 1 PCa and received a Stockholm3 test before surveillance biopsies. The primary outcome was ISUP upgrading, defined as ISUP GG 2 or higher.

**Results:**

At surveillance biopsy, 72 of the 199 (36%) men were upgraded. Men with upgraded PCa had higher median Stockholm3 (35; interquartile range [IQR] 26–50) than those without upgrading (23; IQR 14–34, *P* < 0.01). The area under the receiver operating characteristic curve (AUC) for Stockholm3 was 0.71 (95% confidence interval [CI] 0.64–0.79). For Stockholm3 ≥15, sensitivity, specificity, and negative‐predictive value (NPV) were 0.93 (95% CI 0.85–0.98), 0.26 (95% CI 0.19–0.35), and 0.87 (95% CI 0.72–0.96), respectively, sparing 19% of biopsies while missing five cases (7% of upgrades), none of which were ISUP GG ≥3. At a lower threshold (≥11), sensitivity increased to 0.99 (95% CI 0.93–1.00) with lower specificity (0.11; 95% CI 0.06–0.18) and an NPV of 0.93 (95% CI 0.68–1.00). Prostate‐specific antigen density ≥0.15 showed a sensitivity of 0.60 (95% CI 0.48–0.72), specificity of 0.72 (95% CI 0.63–0.80), and NPV of 0.75 (95% CI 0.66–0.83), with an AUC of 0.71 (95% CI 0.63–0.79). Prostate Imaging‐Reporting and Data System score ≥3 demonstrated a sensitivity of 0.92 (95% CI 0.83–0.97), specificity of 0.33 (95% CI 0.22–0.44), and NPV of 0.84 (95% CI 0.66–0.95), with an AUC of 0.72 (95% CI 0.65–0.79).

**Conclusion:**

In men with low‐risk PCa under AS, Stockholm3 may enhance risk stratification and reduce unnecessary biopsies. Prostate‐specific antigen density and multiparametric magnetic resonance imaging showed comparable performance, with Stockholm3 providing a potentially practical blood‐based complement in a multimodal strategy.

**Abbreviations**
AS: active surveillance
AUC: area under the curve
CI: confidence interval
EAU: European Association of Urology
GG: grade group
IQR: interquartile range
ISUP: International Society of Urological Pathologists
MIC1: macrophage inhibitory cytokine 1
mpMRI: multiparametric magnetic resonance imaging
MSMB: microseminoprotein‐beta
NPV: negative predictive value
PCa: prostate cancer
PI‐RADS: Prostate Imaging‐Reporting and Data System
PPV: positive predictive value
PRECISE: Prostate Cancer Radiological Estimation of Change in Sequential Evaluation
PSA: prostate‐specific antigen
PSAd: prostate‐specific antigen density
PSAdt: prostate‐specific antigen doubling time
ROC: receiver operating characteristic
SNP: single‐nucleotide polymorphism

## Introduction

Prostate cancer (PCa) is one of the most commonly diagnosed cancers among men worldwide, with low‐risk cases often managed through active surveillance (AS) to avoid the potential side effects of radical treatments [1, 2, 3]. AS involves monitoring with prostate‐specific antigen (PSA), digital rectal exams, multiparametric magnetic resonance imaging (mpMRI), and periodic biopsies to detect disease progression requiring treatment. Given the generally indolent nature of low‐risk PCa, this approach balances cancer control and quality of life by reducing overtreatment [4, 5].

Biopsies during AS are essential for monitoring progression and are recommended before transitioning to active treatment [5, 6, 7]. However, their invasive nature, with risks such as bleeding, infection, and discomfort, has driven efforts to reduce their frequency while maintaining accuracy. For years, re‐biopsy and treatment decisions relied on PSA and digital rectal exams. Advances in mpMRI and the Prostate Cancer Radiological Estimation of Change in Sequential Evaluation (PRECISE) classification system have improved detection while reducing unnecessary biopsies [8]. However, even when combined with biomarkers such as PSA density (PSAd) and PSA doubling time (PSAdt), mpMRI cannot fully eliminate biopsies, resulting in a substantial number of potentially avoidable procedures [9].

Given these limitations, less invasive methods are needed to complement current tools, minimizing unnecessary biopsies while ensuring reliable monitoring and reducing uncertainty for physicians and patients [10, 11].

Blood‐based biomarkers, such as the Stockholm3 test, present a promising alternative for enhancing risk stratification in AS. The Stockholm3 test enhances PCa detection and monitoring by integrating clinical variables, blood biomarkers, and genetic markers. The STHLM3 study demonstrated an effective approach to improving the identification of men at higher risk in the primary diagnostic setting for significant PCa [12]. Its effectiveness has been validated across diverse populations and countries, demonstrating its accuracy and ability to reduce reliance on unnecessary mpMRIs and biopsies [12, 13, 14, 15, 16].

The prospective multicentre STHLM3 AS NorDCaP trial explores the predictive abilities of the Stockholm3 test for upgrading among men on AS for low‐risk PCa and its potential role in reducing the need for mpMRI and biopsy in an AS setting.

## Materials and Methods

### Study Design and Population

The STHLM3 AS NorDCaP trial is a prospective, cross‐sectional, non‐comparative, non‐randomized, multicentre study conducted across participating centres in Norway, Denmark, and Sweden; ClinicalTrials.gov identifier: NCT04627948. The study started in July 2019, and the last patient was recruited in December 2022; it aimed to evaluate the predictive value of the Stockholm3 test in identifying PCa upgrading upon re‐biopsy among men undergoing AS.

Eligible participants included men diagnosed with low‐risk PCa, actively enrolled in an AS programme and scheduled for surveillance biopsy as part of their routine clinical follow‐up, including both confirmatory and subsequent surveillance biopsies (Fig. S1). Inclusion criteria were histologically proven localized low‐risk PCa (PSA ≤10 ng/mL; clinical stage ≤T2a, and biopsy International Society of Urological Pathology (ISUP) grade group 1), absence of history of previous PCa treatments, such as surgery, radiation therapy, hormone therapy, or chemotherapy. Sampling and clinical assessments were synchronized with the participants' planned AS follow‐up schedules to avoid unnecessary additional procedures. All participants provided written informed consent.

### Clinical and Diagnostic Assessments

At enrolment, when patients were scheduled for a follow‐up biopsy, a comprehensive baseline assessment was conducted. Peripheral blood samples (12 mL) were collected for calculation of the Stockholm3 risk score. The Stockholm3 test estimates an individual's probability (ranging from 0% to 100%) of harbouring clinically significant PCa (defined as ISUP grade group ≥2) on biopsy. The score was calculated integrating clinical variables (age, first‐degree family history of PCa [father, brother or son], and previous biopsy history), blood biomarkers (total PSA, free PSA, free/total PSA ratio, human kallikrein 2, macrophage inhibitory cytokine‐1, and microseminoprotein beta), and genetic markers (a polygenic risk score based on 254 single‐nucleotide polymorphisms and a specific variable for the HOXB13 single‐nucleotide polymorphism). Additional follow‐up clinical data comprised prostate volume and clinical T‐stage. mpMRI data were collected only if the exam was performed within 3 months before the planned surveillance biopsy, with the Prostate Imaging‐Reporting and Data System (PI‐RADS) v2.1 score used to assess suspicious lesions.

### Patient Follow‐Up and Surveillance Biopsy Protocol

Surveillance biopsies were conducted according to institutional protocols. They were scheduled within 2–6 months after the initial biopsy when used as confirmatory biopsies, or later in the context of AS—either as routine biopsies per institutional guidelines or when a repeat biopsy was deemed necessary because of progression in biomarkers, imaging findings, or digital rectal exam. Biopsies were performed transrectally, with two to four cores taken from each MRI‐visible lesion, and a minimum of 12 systematic cores were also obtained. For each biopsy, the ISUP grade group, cancer length, number of positive cores, and total number of cores were collected.

### Study Outcomes

The primary outcome was upgrading at surveillance biopsy, defined as the detection of ISUP grade group ≥2 PCa at confirmatory or the last surveillance biopsy during AS. Covariates consisted of Stockholm3 score, PSAd, PSAdt, and PI‐RADS score, all determined from the assessment preceding the surveillance biopsy. The PSA value used for analysis served as an input variable for the Stockholm3 score calculation. In a secondary analysis, we applied a more stringent definition of clinically significant disease, defined as ISUP grade group 2 cancers with cancer length >10 mm per targeted core and >10% Gleason pattern 4, as well as ISUP grade group 3–5 disease.

### Sample Size Calculation

Sample size calculations were based on the prevalence of upgrading in AS populations and the expected sensitivity and specificity of the Stockholm3 test. To simulate the expected data, we used data from the STHLM3 study [12], assuming a 20% upgrading rate. We repeatedly sampled datasets of 400 men from the STHLM3 study, where 80% had ISUP grade group 1 cancer and 20% had ISUP grade group ≥2 cancer (representing upgraded cases). Since the Stockholm3 score was available for all men in STHLM3, we could assess its association with upgrading. Based on 1,000 resampled datasets, the power to detect a significant association between the Stockholm3 score and upgrading exceeded 90% with a sample size of 400 men (α = 0.05). The final study population comprised 199 participants, falling short of the target required by the power calculation. The slow recruitment rate was primarily attributed to the onset of the COVID‐19 pandemic, which significantly delayed the enrolment process.

### Statistical Analyses

Descriptive statistics were used to summarize baseline demographic and clinical characteristics, stratified by study centre. Continuous variables were reported as medians with interquartile ranges (IQRs), depending on their distribution, and categorical variables were summarized as frequencies and percentages.

Contingency tables were used to calculate sensitivity, specificity, negative predictive value (NPV), and positive predictive values (PPV) for different Stockholm3 thresholds that have been explored and validated in the primary diagnostic setting, PSAd ≥0.15 and ≥0.10, PSAdt <3 years, and PI‐RADS score ≥3 and ≥4 [17, 18]. The area under the receiver operating characteristic curve (AUC) was calculated for Stockholm3, PSAd, PSAdt, and mpMRI and graphically depicted for Stockholm3, PSAd, and PSAdt.

All statistical analyses were conducted using R statistical software version 4.3.1.

## Results

### Baseline Characteristics

The STHLM3‐AS NorDCaP trial cohort consisted of 199 men under AS, with 61 participants from Denmark, 109 from Norway, and 29 from Sweden. Baseline characteristics are presented in Table 1. The median Stockholm3 was 26 (IQR 17–40). Men with upgraded PCa had a significantly higher median Stockholm3 (35; IQR 26–50) than those without upgrading (23; IQR 14–34, *P* < 0.01) (Fig. S2).

**Table 1:** Clinical characteristics of the 199 men enrolled in the STHLM3 active surveillance NorDCaP trial.

Variables	Denmark	Norway	Sweden
Total number of patients	*N* = 61	*N* = 109	*N* = 29
Age, years	69 (64–73)	66 (62–70)	67 (62–72)
Stockholm3 score	25 (19–37)	28 (17–40)	30 (15–31)
PSA, ng/mL	6.3 (4.6–8.5)	5.2 (2.7–7.8)	4.3 (3.7–8.5)
PSA at diagnosis, ng/mL	6.8 (5.8–8.1)	4.3 (3.0–5.8)	4.3 (3.1–5.6)
PSA density	0.12 (0.08–0.17)	0.14 (0.08–0.20)	0.11 (0.09–0.16)
PSA doubling time, months	−8.9 (−24.6–22.3)	25.3 (−32.9–76.3)	36.6 (8.7–89.1)
Time under surveillance, months	9.7 (4.4–14.2)	15.8 (13.5–28.7)	16.7 (9.7–34.8)
Missing PSA doubling time	1 (2)	5 (5)	0 (0%)
Abnormal DRE	NA	5 (5)	0 (0%)
Missing DRE	NA^a^	14 (13)	19 (7%)
Family history of prostate cancer	13 (23)	42 (40)	7 (25%)
Missing family history	5 (8)	3 (3)	1 (3%)
Previous negative biopsy	54 (95)	107 (98)	23 (85%)
Missing previous negative biopsy	4 (7)	0 (0)	2 (7%)
MRI performed	61 (100)	104 (95)	29 (100%)
PI‐RADS ≤2	26 (43)	45 (43)	10 (34%)
PI‐RADS 3	2 (3)	23 (22)	9 (31%)
PI‐RADS 4	16 (26)	27 (26)	9 (31%)
PI‐RADS 5	17 (28)	9 (9)	1 (3%)
Benign biopsy	15 (25)	20 (18)	6 (21%)
ISUP 1	32 (52)	42 (39)	12 (41%)
ISUP 2	13 (21)	35 (32)	10 (34%)
‐% Gleason pattern 4	5 (5–5)	10 (10–20)	13 (11.5–20)
Missing data on % Gleason pattern 4	12	2	3
ISUP 3–5	1 (2)	12 (11)	1 (3%)
Length of cancer in biopsy			
<5 mm	NA	26 (29)	NA
5–9.9 mm	NA	18 (20)	NA
≥10 mm	NA	37 (42)	NA
Missing	46 (100)	9 (10)	23 (100%)

Data are presented as median (interquartile range) or n (%) unless otherwise indicated.

^a^ DRE data were not consistently available in Danish centres and are therefore not reported.

DRE, digital rectal examination; ISUP, International Society of Urological Pathologists; MRI, magnetic resonance imaging; NA, not applicable; PI‐RADS, Prostate Imaging‐Reporting and Data System; PSA, prostate‐specific antigen.

**Fig. 1:**
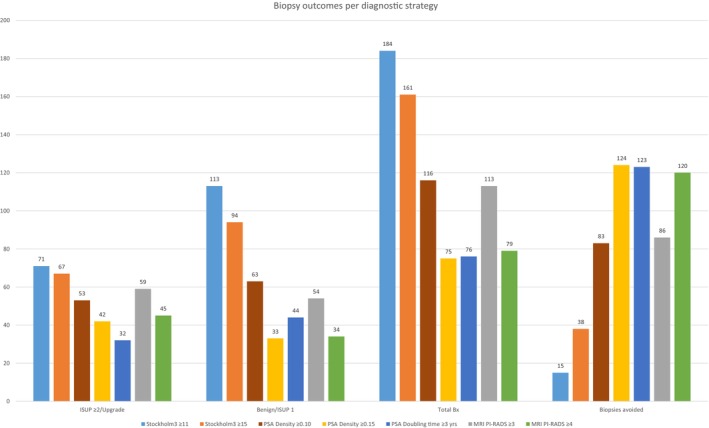
Biopsy outcomes according to the diagnostic strategy using Stockholm3, prostate‐specific antigen (PSA) density, PSA doubling time, and magnetic resonance imaging (MRI) if they would have been used to indicate that men under active surveillance should undergo biopsy. For each diagnostic strategy and threshold, the figure shows the number of patients with upgrading to International Society of Urological Pathologists (ISUP) grade group ≥2, benign/ISUP 1 findings, and the number of biopsies that could have been avoided. The ‘total biopsies’ group represents the number of biopsies that would have been performed under each strategy. MRI, magnetic resonance imaging; PI‐RADS, Prostate Imaging‐Reporting and Data System; y, years.

Biopsy results showed that 21% (41/199) had benign findings, 43% (86/199) had ISUP grade group 1 cancer, 29% (58/199) had ISUP grade group 2 cancer, and 7% (14/199) had ISUP grade group 3–5 PCa (Fig. 1). No significant differences were observed across the three centres, except for PSAdt.

### Performance of Various Tests in Predicting PCa Upgrading

The performance characteristics of the tests predicting PCa upgrading are summarized in Table 2, with full contingency data for all thresholds provided in Table S1. The AUC of Stockholm3 for predicting upgrading was 0.71 (95% confidence interval [CI] 0.64–0.79). At the prespecified threshold of ≥11, sensitivity was 0.99 (95% CI 0.93–1.00), specificity 0.11 (95% CI 0.06–0.18), PPV 0.39 (95% CI 0.32–0.46), and NPV 0.93 (95% CI 0.68–1.00). When the Stockholm3 threshold was increased to ≥15, sensitivity decreased to 0.93 (95% CI 0.85–0.98), whereas specificity improved to 0.26 (95% CI 0.19–0.35), sparing 19% of biopsies while missing five cases (7% of upgrades), none of which were ISUP grade group ≥3. The PPV was 0.42 (95% CI 0.34–0.50), and the NPV was 0.87 (95% CI 0.72–0.96). At a threshold of ≥11, 1 of 72 upgrading events (1.4%) would have been missed, whereas 14 of 199 biopsies (7%) could have been avoided. In contrast, at a threshold of ≥15, 38 of 199 biopsies (19%) could have been avoided, whereas 5 of 72 upgrading events (7%) would have been missed; importantly, none of the missed cases were ISUP grade group ≥3.

**Table 2:** Sensitivity, specificity, positive predictive value, negative predictive value, and area under the receiver operating characteristic curve (AUC) of the various tests predicting prostate cancer upgrading at re‐biopsy.

Biomarker/test	Threshold	Sensitivity (95% CI)	Specificity (95% CI)	AUC (95% CI)	PPV (95% CI)	NPV (95% CI)
Stockholm3	≥11	0.99 (0.93–1.00)	0.11 (0.06–0.18)	0.71 (0.64–0.79)	0.39 (0.32–0.46)	0.93 (0.68–1.00)
	≥15	0.93 (0.85–0.98)	0.26 (0.19–0.35)		0.42 (0.34–0.50)	0.87 (0.72–0.96)
PSA density	≥0.10	0.76 (0.64–0.85)	0.46 (0.37–0.56)	0.71 (0.63–0.79)	0.46 (0.36–0.55)	0.76 (0.65–0.86)
	≥0.15	0.60 (0.48–0.72)	0.72 (0.63–0.80)		0.56 (0.44–0.67)	0.75 (0.66–0.83)
PSA doubling time	<3 years	0.54 (0.42–0.66)	0.36 (0.27–0.45)	0.56 (0.48–0.64)	0.32 (0.24–0.42)	0.58 (0.46–0.69)
MRI	PI‐RADS ≥3	0.92 (0.83–0.97)	0.33 (0.22–0.44)	0.72 (0.65–0.79)	0.52 (0.43–0.62)	0.84 (0.66–0.95)
	PI‐RADS ≥4	0.70 (0.58–0.81)	0.58 (0.46–0.68)		0.57 (0.45–0.68)	0.71 (0.58–0.81)

CI, confidence interval; MRI, magnetic resonance imaging; NPV, negative predictive value; PI‐RADS, Prostate Imaging‐Reporting and Data System; PPV, positive predictive value; PSA, prostate‐specific antigen.

**Fig. 2:**
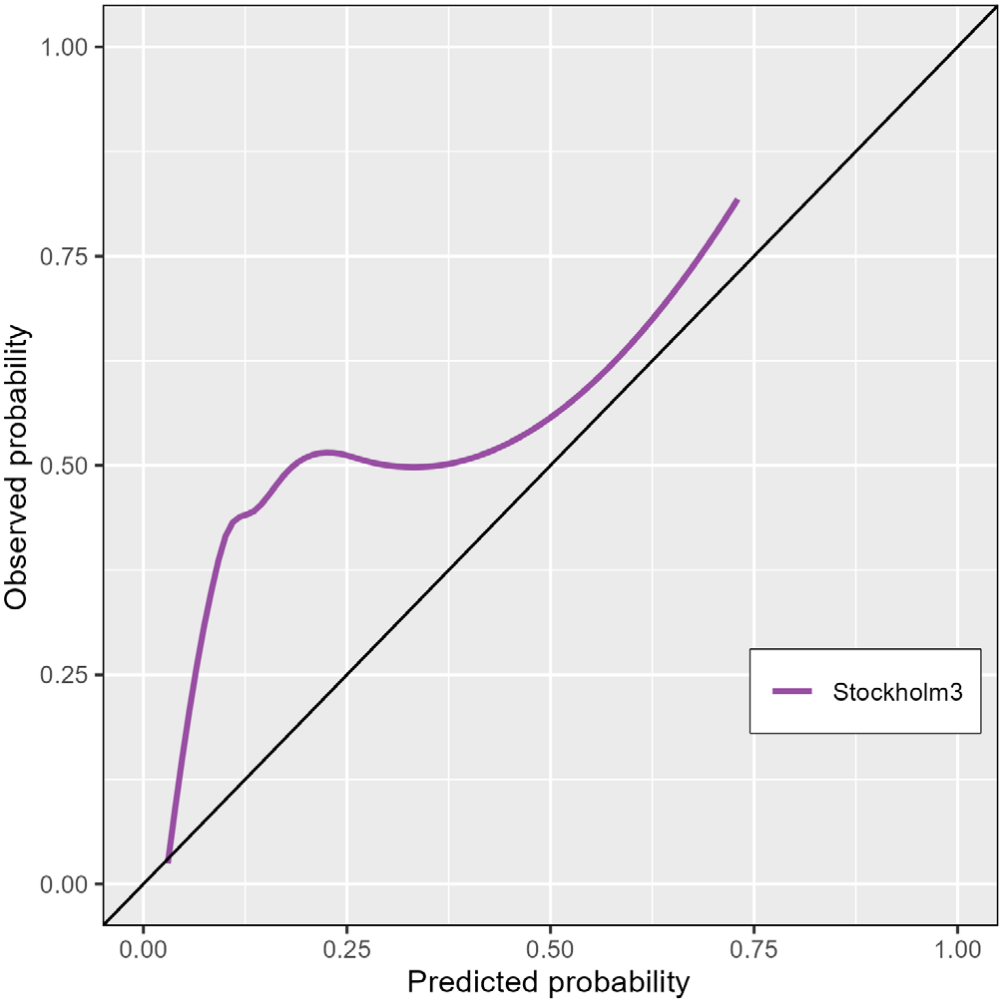
Calibration plot for Stockholm3 for International Society of Urological Pathologists grade group ≥2 prostate cancer (PCa).

The calibration plot for Stockholm3, showing expected versus observed upgrading for ISUP ≥2 PCa, is provided in Fig. 2. Visual inspection of the calibration plot suggests imperfect calibration, with systematic underestimation of upgrading risk, particularly in the lower to intermediate predicted probability range. This indicates that its absolute risk estimates may be less reliable in this cohort, as previously observed in validation studies in the primary diagnostic setting [18, 19, 20].

Exploratory analyses using a more stringent definition of higher‐risk disease (defined as ISUP grade group 2 cancers with cancer length >10 mm per targeted core and >10% Gleason pattern 4, as well as ISUP grade group 3–5 disease) showed broadly comparable results across all tests (see Appendix S2, Table S2). In this context, Stockholm3 maintained moderate discriminative performance, with a similar trade‐off between sensitivity and specificity across thresholds, supporting the robustness of its performance in identifying clinically more aggressive disease. However, this subgroup analysis is likely underpowered because of the small sample size (*n* = 25) and should therefore be interpreted with caution.

**Fig. 3:**
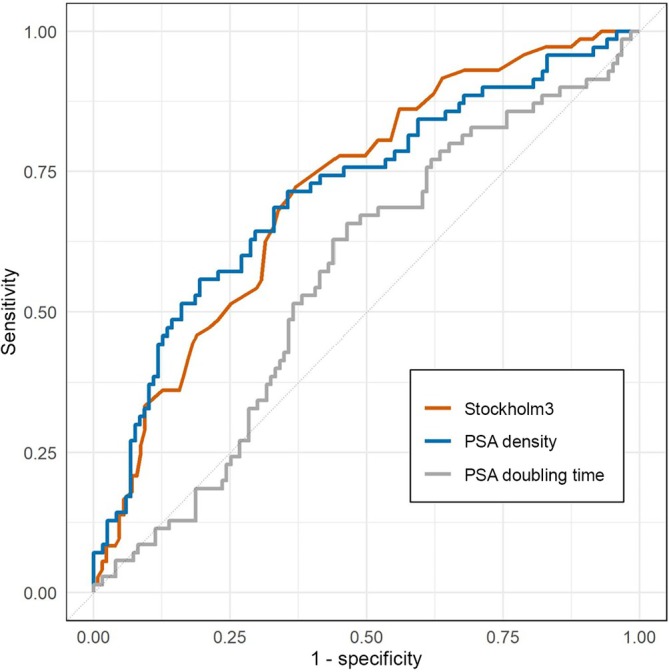
Receiver operating characteristic curve for Stockholm3, prostate‐specific antigen (PSA) density and PSA doubling time for the total cohort of men defining the outcome as International Society of Urological Pathologists grade group ≥2 prostate cancer on biopsy.

The diagnostic performance of PSAd, mpMRI, and PSAdt is summarized in Table 2. The receiver operating characteristic curves of Stockholm3, PSAd, and PSAdt for upgrading to ISUP grade group ≥2 PCa at re‐biopsy are depicted in Fig. 3.

## Discussion

The STHLM3 AS NorDCaP trial assessed the accuracy of the Stockholm3 test in predicting PCa upgrading among men on AS for low‐risk PCa. Stockholm3 performed well across different thresholds, with high sensitivity and NPV at ≥11 and improved specificity at higher thresholds (≥15). Its performance was comparable to that of PSAd and mpMRI, with similar AUC values across all three tests. Diagnostic accuracy varied with threshold selection, underscoring the importance of aligning cutpoints with clinical priorities. Stockholm3 shows potential in reducing unnecessary surveillance biopsies while maintaining reliable detection of cancer upgrading.

The first evaluation of Stockholm3 in the AS setting was performed by Olsson et al. in the STHLM3‐AS trial [21]. Using a >10% cutoff for risk of Gleason ≥3 + 4 cancer, they demonstrated that excluding Stockholm3‐negative men could reduce biopsies by 56.8% and MRI examinations by 22.5%, while missing only 7.2% of GS ≥3 + 4 cancers. This study was restricted to a Swedish cohort (*n* = 280). Our study builds on these findings in a multinational Nordic cohort and explores multiple cutpoints. Taken together, both studies indicate that Stockholm3 can potentially reduce unnecessary investigations with only a modest risk of missed upgrading. Our findings emphasize the role of threshold selection: a lower cutoff maximizes sensitivity, whereas a higher cutoff spares more biopsies but misses upgrades. Unlike Olsson et al., our study extends validation beyond Sweden and provides explicit confidence intervals, thereby adding evidence on the potential role of the Stockholm3 test in the AS setting.

AS is recognized as the standard of care to manage low‐risk PCa, avoiding overtreatment and minimizing side effects while monitoring disease progression. The European Association of Urology guidelines for AS recommend a strict protocol involving at least annual digital rectal examinations, PSA testing every 6 months, and repeat biopsies every 2–3 years [6]. However, patients with low‐risk PCa who have stable mpMRI findings and a low PSAd (<0.15) may be exempt from repeat biopsies [6]. Currently, PCa surveillance heavily relies on these clinical tools. According to two meta‐analyses, mpMRI progression during AS showed value in predicting histological progression. Both studies reported a pooled histological progression rate of 27%. Triggering biopsies solely based on mpMRI progression would miss about 40% of men with histological progression. Both studies concluded that serial MRI should not be relied upon as the sole factor for excluding PCa progression during AS [22, 23].

During active surveillance, monitoring tumour progression to avoid missing a window of curative treatment is a key priority for physicians and many patients. At the same time, reducing unnecessary biopsies and mpMRI is an important goal for others, reflecting heterogeneity in patient preferences. Although some men prioritize minimizing the risk of disease progression, others strongly value avoidance of invasive procedures, highlighting an important individual dimension in how priorities are perceived during AS [24]. Therefore, the selection of a test and its threshold should balance oncological safety with the desire to minimize invasiveness, aiming to maintain high sensitivity and NPV while acknowledging potential trade‐offs in specificity. Our results demonstrated that the Stockholm3 test performed well across different thresholds, with a high sensitivity and NPV at lower thresholds (≥11) and better specificity at higher thresholds (≥15). Thus, at a threshold of ≥11, the Stockholm3 test demonstrated a sensitivity of 0.99 (95% CI 0.93–1.00) and an NPV of 0.93 (95% CI 0.68–1.00), indicating that it would have missed only 1 of 72 (1.4%) patients with pathologically confirmed upgrading. By using this threshold, unnecessary biopsies would have been performed in 113 of 184 cases (61%). In contrast, PSAd (≥0.15), PSAdt (<3 years), and mpMRI (PI‐RADS ≥3), using their recommended cutpoints, demonstrated lower sensitivities of 0.60 (95% CI 0.48–0.72), 0.54 (95% CI 0.42–0.66), and 0.92 (95% CI 0.83–0.97), respectively. The observed miscalibration suggests that absolute risk estimates should be interpreted with caution, as underestimation of upgrading risk could potentially lead to inappropriate deferral of biopsy in some patients; further studies in the AS setting are warranted to better characterize calibration performance. Furthermore, PSAd, PSAdt, and mpMRI would have led to unnecessary biopsies in 33/75 (44%), 79/117 (68%) and 54/113 (48%) of cases, respectively, where no upgrading was observed, contributing to a significant number of unnecessary procedures and associated costs. When applying a lower PSAd threshold of ≥0.10, sensitivity increased to 0.75 (95% CI 0.64–0.85) at the expense of reduced specificity (0.46; 95% CI 0.37–0.56), reflecting the expected trade‐off between improved detection of upgrading and a higher number of unnecessary biopsies. The corresponding PPV and NPV were 0.46 (95% CI 0.36–0.55) and 0.76 (95% CI 0.65–0.86), respectively, with an AUC of 0.71 (95% CI 0.63–0.79).

According to our results, the currently recommended cutpoints for PSAd to trigger re‐biopsy during AS may miss significant cancer upgrades when used alone, highlighting the need for a multimodal approach in this setting. Stockholm3 offers higher sensitivity and negative predictive value at the suggested cut‐off, reducing the risk of missing significant upgrades and potentially avoiding unnecessary biopsies. Incorporating it into follow‐up strategies could improve the safety and efficiency of monitoring [6]. To better align with current clinical practice, we assessed the ability of combining Stockholm3, PSAd, and PSAdt to predict upgrading in patients undergoing AS follow‐up without relying on mpMRI. Among the 72 of 199 patients (36%) who experienced upgrading on re‐biopsy, using the recommended cutpoints for PSAd (≥0.15) and PSAdt (<3 years), together with either Stockholm3 threshold (≥11 or ≥15), at least one of these three tests would have triggered a re‐biopsy in all upgraded cases. This indicates that, independent of the Stockholm3 cutoff chosen, the combined strategy identified all upgrades.

Both PSAd and mpMRI demonstrated overall diagnostic performance comparable to Stockholm3 in predicting upgrading, with similar discriminative ability, particularly for PSAd, which is a widely available and inexpensive tool (AUC 0.71 [95% CI 0.63–0.79]). mpMRI showed a similarly comparable performance (AUC 0.72 [95% CI 0.65–0.79]) but remains more resource intensive and less universally accessible. In this context, Stockholm3 did not outperform MRI but offers a practical, blood‐based alternative with high sensitivity and NPV at selected thresholds, supporting its potential role as part of a multimodal risk‐stratification strategy rather than a standalone replacement.

In summary, introducing the Stockholm3 test into AS protocols may enhance risk stratification and decrease the need for repeat biopsies and mpMRI in patients with stable disease, ultimately minimizing reliance on invasive procedures.

Despite this promising potential, the study has several limitations. First, the sample size calculated at the outset to achieve the prespecified power was not met because of delays in recruitment caused by the COVID‐19 pandemic. As a result, only about 50% of the target sample was recruited. This limitation may affect the statistical power of the study and potentially limit the robustness of the findings. A smaller sample size can also reduce the precision of the estimates and the ability to detect subtle effects, making the results more susceptible to variability. However, the multinational approach is a strength, as it provides a more diverse and representative sample, enhancing the applicability of the results across different populations. Additionally, although the Stockholm3 test demonstrated high sensitivity and NPV, its low specificity at certain thresholds, such as ≥11, could still lead to a high number of benign biopsies. Furthermore, progression on mpMRI as defined by PRECISE criteria, is known to be a strong predictor of histological upgrading [22, 25]. Although mpMRI was used in the study, it was classified according to the PI‐RADS system, and a comparative analysis using the PRECISE criteria is lacking. Furthermore, data on biopsy setting (confirmatory vs surveillance) was not systematically collected, which may have influenced upgrading risk estimates and precluded stratified analyses. In addition, the cohort consisted exclusively of men with ISUP grade group 1 disease, and mpMRI was not systematically performed at the time of initial diagnosis, as patient inclusion started in 2019 when MRI was not yet recommended as a standard diagnostic tool. As such, the findings may not fully generalize to contemporary AS cohorts that include more patients with ISUP grade group 2 and MRI‐visible disease. Furthermore, the cross‐sectional and non‐randomized design of the study further limits the ability to draw conclusions about longitudinal safety and the true predictive accuracy of the Stockholm3 test over time. Finally, the long‐term impact of incorporating the Stockholm3 test into clinical practice remains uncertain and requires further validation in larger, ethnically diverse populations. Economic implications, including test costs and potential savings from avoided procedures, also warrant formal evaluation in future cost‐effectiveness studies.

In summary, the results of our study demonstrate that, in men with low‐risk PCa undergoing AS, the Stockholm3 test shows potential to improve risk stratification and reduce unnecessary biopsies. PSA density and mpMRI performed similarly to Stockholm3 for predicting upgrading; however, Stockholm3 offers a practical blood‐based complement within a multimodal risk‐stratification strategy, indicating its potential to refine active surveillance protocols compared with traditional diagnostic methods. However, further validation in larger, more diverse populations is needed to establish its long‐term clinical utility for patients undergoing AS.

## Author Contributions

NG, SS, TP, AL, TN, ME, and HG contributed to study conception and design. EFJ, MB, BGP, KDS, SRK, and AL contributed to data acquisition. NG, SS, TP, and AL contributed to data analysis and interpretation. NG and SS drafted the manuscript. TP, TN, ME, HG, EFJ, MB, BGP, KDS, SRK, and AL critically revised the manuscript for important intellectual content. TP performed statistical analysis. SRK, BGP, and KDS obtained funding. All authors reviewed the manuscript, approved the final version, and agree to be accountable for the integrity and accuracy of the work.

## Disclosure of Interests

Anna Lantz has received speaker honoraria from Janssen, Ipsen, Johnson & Johnson, Bayer, and Accord Healthcare AB. She is the site principal investigator for the EMBARK trial at Karolinska University Hospital (site 723; MDV3100‐13), a study funded by Pfizer and Astellas Pharma. Anna Lantz serves as section editor for prostate cancer at *BJU International* and had no role in the editorial handling or peer review of this manuscript. She is the medical lead for Organized Prostate Cancer Testing in region Stockholm and a member of the steering group of the Swedish Register for Organized Prostate Cancer Testing. She has conducted research evaluating the Stockholm3 test within the OPT programme (unpublished). Anna Lantz reports no personal financial interests related to the Stockholm3 test. Karina D Sørensen is a co‐inventor on patents for microRNA and DNA methylation markers for PCa, owned by Aarhus University and Aarhus University Hospital. The Karolinska Institutet collaborates with A3P Biomedical in developing the technology for the Stockholm3 test. Martin Eklund reports ownership of stock in A3P Biomedical AB and Clinsight AB, holds patents related to PCa risk prediction, and has received honoraria from Johnson & Johnson, Astellas, and Ipsen. Tobias Nordstrom reports owning shares in A3P Biomedical. Thorgerdur Palsdottir is employed by A3P Biomedical. Henrik Grönberg report owning shares in A3P Biomedical. He reports four pending PCa diagnostic‐related patents: method for indicating a presence or non‐presence of aggressive PCa (WO2013EP7425920131120); prognostic method for individuals with PCa (WO2013EP7427020131120); method for indicating a presence of PCa in individuals with particular characteristics (WO2018EP5247320180201); and method for indicating the presence or non‐presence of PCa (WO2013SE5055420130516). Henrik Grönberg reports one additional pending PCa diagnostic‐related patent: method for detecting solid tumour cancer (WO2015SE5027220150311). The remaining authors declare no conflicts of interest.

## Funding

Dr Kjosavik reports receiving a grant by the Norwegian Cancer Society and The Norwegian Prostate Cancer Association (grant no. 197950). Dr Dalsgaard Sørensen and Dr Bodil Ginnerup Pedersen report receiving a grant by Innovation Fund Denmark & Nordforsk (grant no. 8114‐00014B, granted to Dr Dalsgaard Sørensen) and by The Danish Cancer Society (R167‐A10920 granted to Dr Dalsgaard Sørensen; and R175‐A11419 grated to Dr Bodil Ginnerup Pedersen).

## Ethics Approval

The study was approved by the Swedish Ethical Review Authority (Etikprövningsmyndigheten) under approval number Dnr 2019‐02020. All patients provided written informed consent before inclusion. The study was designed and conducted in accordance with the principles of the Declaration of Helsinki.

## Supporting information


**Fig. S1.** Study design.
**Fig. S2.** Boxplot of Stockholm3 and prostate‐specific antigen (PSA) for the active surveillance study cohort stratified by upgrade on biopsy.
**Table S1.** Contingency tables and diagnostic performance of Stockholm3 and comparator markers for prediction of upgrading to International Society of Urological Pathologists grade group ≥2.
**Table S2.** Sensitivity, specificity, positive predictive value, negative predictive value, and area under the receiver operating characteristic curve (AUC) of the various tests for predicting prostate cancer upgrading at re‐biopsy in high‐risk disease (defined as International Society of Urological Pathologists grade group ≥3 or 2 with cancer length ≥10 mm and/or >10% Gleason pattern 4).

**Appendix S2.** Supporting Information.

## Data Availability

Data cannot be shared for ethical/privacy reasons. Data will be made available to researchers who provide a methodologically robust proposal in which the aims are relevant and clear. Proposals can be submitted up to 24 months after publication of the article and should be directed to anna.lantz@regionstockholm.se. Only proposals with ethical approval will be granted access to the data. Please contact anna.lantz@regionstockholm.se with requests.
