# Supplementary material for: STHLM3 NorDCaP: prospective multicentre trial of Stockholm3 for active surveillance biopsy guidance
Source: BJU Int. 2026 Jul 1;138(3):471–9. doi: 10.1111/bju.70366 (PMC13458658; doi:10.1111/bju.70366)
Supplement: Supplementary file 1 [file BJU-138-471-s002.pdf]

Study Protocol

STHLM3 AS NorDCaP

Principal Investigator

Anna Lantz, MD, PhD, Karolinska Institutet

Co-investigators in alphabetical order

Martin Bergman, MD, Capio S:t Göran Hospital, Sweden

Martin Eklund, PhD, Karolinska Institutet, Sweden

Karina Dalsgaard Sørensen, PhD, Aarhus University Hospital, Denmark

Kristina Hotakainen, MD, PhD, Mehiläinen, Finland

Svein Kjosavik, MD, PhD, Stavanger University Hospital, Norway

January 2019, version 1

Karolinska Institutet

HELSE STAVANGER
Stavanger universitetssjukehus

AARHUS UNIVERSITET

MEHILÄINEN

Capio S:t Görans Sjukhus

Table of contents

1 Abstract 3
2 Background 4
2.1 Public health significance of prostate cancer 4
2.2 Early detection of prostate cancer: benefits and harms 4
2.3 Active surveillance 4
2.4 The STHLM3 AS study 5
3 Scientific aims 6
3.1 Primary aim 6
3.2 Primary endpoint 6
3.3 Secondary aims 6
4 Study design 6
4.1 Study size 6
4.2 Participation criteria 7
4.2.1 Inclusion criteria 7
4.2.2 Exclusion criteria 7
5 Statistical analysis plan 7
5.1 Objective 7
5.2 Analysis 7
5.2.1 Show association for Stockholm3 and upgrading 7
5.2.2 Compare association between Stockholm3 and PSA for upgrading 7
5.2.3 Show sensitivity versus specificity for Stockholm3 and upgrading 7
5.3 Power 7
6 Study process 7
6.1 Step 1: Selection and Invitation 7
6.2 Step 2: Blood Sampling 8
6.3 STEP 3: Follow-up 8
6.4 Step 4: Data collection 8
7 Ethical considerations 8
7.1 Is there an increased risk of missing aggressive cancers? 8
7.2 Is there a risk of over-treatment? 8
7.3 How do we protect the personal integrity? 8
8 Timeline 9
9 References 9

1 Abstract

Prostate cancer is the leading cause of cancer death among men in the Western world. Early detection of prostate cancer has been shown to decrease mortality but has limitations with low specificity leading to unnecessary biopsies, over-diagnosis, and over-treatment of indolent cancers.

Conservative management with active surveillance (AS)[1] is increasingly utilized for low-risk prostate cancers to delay or avoid aggressive treatment and potential side effects. Active surveillance includes serial testing for disease progression to offer selective delayed treatment with curative intent. In Sweden today, the national guidelines prescribe a follow-up for these patients including regular PSA tests and prostate biopsies. Urosepsis is the most feared complication of prostate biopsies. The incidence is around 2% and if left untreated will be life threatening.

The STHLM3 trial has paved the way for improved specificity in early detection of prostate cancer using the blood-based Stockholm3 test for identifying men at increased risk of harboring significant prostate cancer (defined as ISUP ≥ 2). A follow-up of men on AS with Stockholm3 could potentially increase the detection of clinically significant cancers while reducing the rate of prostate biopsies for this group of men with a life-long cancer follow-up. The ongoing *STHLM3 AS* study has shown promising preliminary results in using Stockholm3 to predict upgrading in men on AS.

The aim of the *STHLM3 AS NorDCaP* study is to validate the preliminary results from the *STHLM3 AS* study, i.e. that the Stockholm3 test can be used in an AS cohort to predict upgrading. The study follows a paired design, evaluating our proposed protocol for follow up men on Active Surveillance with Stockholm3 versus the standard protocol according to [country] National Guidelines with PSA.

2 Background

2.1 Public health significance of prostate cancer

Prostate cancer is the most common cancer and the leading cause of cancer death among men in Sweden. In 2017 approximately 10 000 men were diagnosed with prostate cancer and more than 2 500 died due to the disease. With over 90 000 prevalent cases, the health burden and the costs on the health care system are substantial. While a number of risk factors have been proposed for prevention of prostate cancer, including diet and occupational exposures, the only factors conclusively shown to increase risk of the disease are age, ethnicity and family history. Given the high prevalence of the cancer and limited opportunities for primary prevention, improved detection would reduce both procedure-related harm to men and economical cost in the healthcare system.

2.2 Early detection of prostate cancer: benefits and harms

The PSA test was first used to monitor disease progression in prostate cancer patients. However, the PSA test was quickly also used as a *de facto* screening test for prostate cancer in many countries, leading to rapid rises in prostate cancer incidence. The test characteristics for the PSA test in detecting prostate cancer have a sensitivity of 72% and a specificity of 30-35% at a test threshold of 4 ng/ml[2]. However, a lower threshold of 3 ng/ml adopted in Sweden has led to increased sensitivity at the expense of reduced specificity. Results from the large European Randomised Study of Screening for Prostate Cancer (ERSPC) including over 180,000 men provide increasing evidence that PSA screening has led to reduced mortality. This report showed that PSA screening without digital rectal examination was associated with significant relative reduction in prostate cancer mortality when comparing the screening group and control group of 21% and 27% in men who participated in the study at the follow-up of 13 years. Estimations from the ERSPC trial (men aged 55-69) show that 781 men would need to be offered screening and an additional 27 would need to be managed to prevent one prostate-cancer death during a 10-year period, leading to a significant overtreatment of indolent disease[3]. The effectiveness of PSA testing was more marked at the Göteborg site of the ERSPC trial, with a risk reduction of 44% over 14 years in men aged 50-64[4]. This effect size is larger than that observed for mammographic screening for breast cancer and fecal occult blood testing for colorectal cancer.

However, using traditional systematic biopsies for diagnosis, approximately half of diagnosed cancers are low-risk tumors using the same main cutoff for biopsy as the ERSPC trial (PSA=3ng/ml)[5,6]. It has been shown that men with low-risk tumors treated without curative intent have the same survival as men in the background population[7], illustrating the large proportion of over-diagnosed cancers[8].

The STHLM3 study has shown a way to improve identification of men at increased risk of significant prostate cancer. Using the Stockholm3 test, 32% of the prostate biopsies may be saved while not decreasing the sensitivity to high-grade disease (defined as ISUP ≥ 2) and simultaneously decreasing the number of low-grade tumors (ISUP 1) by 17%, thus decreasing overdiagnosis[6].

Men at increased risk of prostate cancer - commonly estimated using PSA and palpatory findings - are traditionally assessed using systematic prostate biopsies. The procedure is performed under local anesthesia using antibiotic prophylaxis and includes 10-12 cores taken from predefined areas of the peripheral zone of the gland as visualized by endorectal ultrasound. While the biopsies systematically cover the prostatic gland rather than targeting a lesion, and non-lethal tumors are common, the risk of over-diagnosis (i.e. detection of non-significant tumors) is high[8]. The risk of severe post-biopsy infection has increased to 2% with increasing frequency of antibiotic resistance, further illustrating the need to decrease the number of performed biopsies[10].

2.3 Active surveillance

At the time of diagnosis not all prostate cancers are suitable for curative treatment. There has been a substantial increase in the incidence of prostate cancer since the introduction of PSA and it has contributed to the detection of many low-risk, clinically insignificant cancers. It has been shown in studies that most men who die within 10-15 years after a diagnosis of low or intermediate risk prostate cancer will die from causes other than from the cancer itself [11,12].

Some reports have shown over-diagnosis in as much as 50% or more in older men[13]. In a randomized controlled trial by Wilt et al. radical prostatectomy did not significantly reduce all-cause or prostate cancer mortality compared with observation after 12 years of follow-up among men with localized prostate cancer detected during the early era of PSA testing[14]. However, radical prostatectomy was associated with reduced all-cause mortality among men with a PSA > 10 ng/mL and possibly among those with intermediate-risk or high-risk tumors. Furthermore, there is uncertainty as to whether Gleason grade 3 has the ability to metastasize, and if this is the case, the question remains whether such cancers should be treated at all[15,16]. However, there is a correlation between the propagation of Gleason grade 3 and the risk for simultaneous Gleason grade 4 cancer[17]. Considering all the evidence, there is a place for active surveillance in selected cases of prostate cancer.

In Sweden, active surveillance is recommended for patients with very low risk prostate cancer and an expected survival of 10 years or more[18]. This is to avoid overtreatment, as well as the potential side effects the treatment causes. The longer expected survival of the patient, the more important it is to perform a thorough investigation to exclude the possibility of a more aggressive cancer before committing a patient to active surveillance, since about 30 percent of all Gleason 3+3 in biopsies are upgraded to a higher Gleason grade after surgery due to sampling error when using systematic biopsies[19]. Based on this, about one third of all patients are understaged on their initial prostate biopsy, and one biopsy alone is not sufficient to offer the patient active surveillance as an alternative[20]. Therefore, a re-biopsy has to be performed before commencing active surveillance. Active surveillance should not be mistaken for watchful waiting, which is the monitoring of a patient until he develops metastatic disease that requires palliative treatment.

Active surveillance according to Swedish National Guidelines consists of:

- a new biopsy with 10 cores within 2-6 months after the initial biopsy, preferably in the anterior part of the prostate[21]
- PSA repeated every 3-4 month
- DRE repeated every 6-8 months for 2 years
- PSA and DRE repeated every 6 months after the initial 2 years of active surveillance
- re-biopsy every 2-3 years including 10 cores
- optional re-evaluation of the primary pathology report
- optional multi-parametric MRI

The longer the expected survival of the patient, the lower the threshold for recommending treatment if monitoring shows any signs of more aggressive cancer. Active surveillance is not optimal for a patient with anterior cancer, a large prostate, a PSA ratio <0.1, prostatitis/UTI or family history of prostate cancer[18]. Active surveillance can induce patient anxiety, as it causes the patient to live with an untreated cancer. This in itself may be a reason to initiate treatment[22,23]. A potential downside of recommending active surveillance to men with low-risk cancers that are still curable is that such cancers could progress over time where potentially the opportunity for curative treatment could be missed. Furthermore, multiple biopsies could complicate future attempts at nerve-sparing surgery through potential distortion of the anatomy.

Active surveillance ends when curative treatment is started or when the patient is no longer a candidate for curative treatment (e.g. due to old age or comorbidity) and thus converts to watchful waiting, or when death occurs.

2.4 The STHLM3 AS study

The STHLM3 AS study is a forerunner to the STHLM3 AS NorDCaP study. The STHLM3 AS study is currently ongoing – recruiting former STHLM3-participants on AS in Stockholm. Preliminary results from the STHLM3 AS study show an association between the Stockholm3 test and upgrading on rebiopsy.

3 Scientific aims

Active surveillance (AS) is an important option to reduce overtreatment of low-risk prostate cancers that are unlikely to cause symptoms if left untreated. However, some tumors with initial low-risk features will develop into lethal disease and the intent of AS is to filter out these men in order not to miss the time window of opportunity for cure. The recommendations from EAU guidelines are to follow men on AS with serial PSA measurements and repeated systematic biopsies, which in 2% causes severe post-biopsy infections.

The hypothesis of the proposed research is that a new protocol for surveillance of low-risk prostate cancers with the Stockholm 3 test could provide equally safe monitoring as the conventional protocol with PSA, but minimizing the numbers of prostate biopsies and thus demonstrate superior specificity and noninferior sensitivity in comparison to the conventional protocol.

3.1 Primary aim

Validate the preliminary results from the STHLM3 AS study, i.e. that the Stockholm3 test can be used in an AS cohort to predict upgrading.

3.2 Primary endpoint

Number of ISUP ≥ 2 disease

3.3 Secondary aims

Compare the association to predict upgrading between Stockholm3 and PSA

4 Study design

STHLM3 AS NorDCaP is a cross-sectional multi-center study, evaluating the predictive value of Stockholm3 test in a prostate cancer cohort on AS for upgrading on rebiopsy. Men from the study sites, diagnosed with prostate cancer and currently on AS and planned for rebiopsies will be invited to the study. Eligible individuals have to be alive without any severe comorbidity and without a history of initiating treatment; surgery, radiation, hormone therapy or chemotherapy. To avoid the need for additional investigations, sampling will be synchronized with timing for planned follow-up within the AS program for each individual.

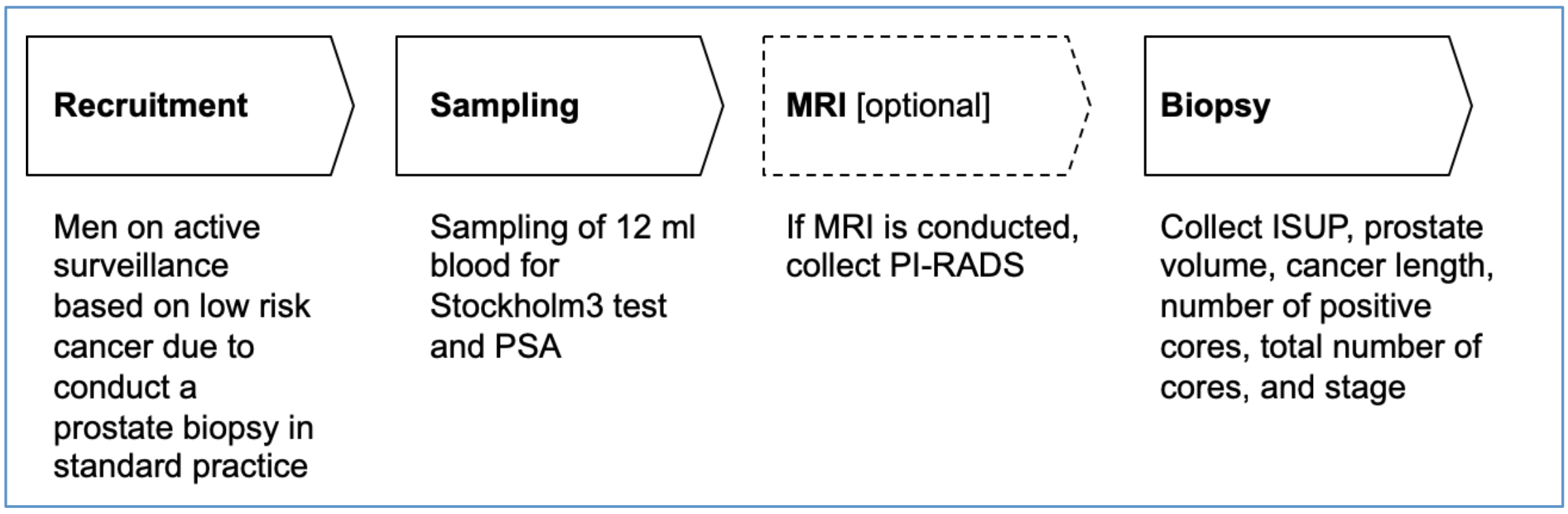

At baseline blood will be drawn for Stockholm3 analyses. The patient will be asked to fill out an informed consent. For each man the following data will be collected: Stockholm3 Risk Score, Stockholm3 Prostate Volume cut-off, total PSA, DRE status, MRI results (PI-RADS, optional, only if MRI is conducted within 3 months before biopsy), prostate volume, biopsy results (ISUP, cancer length, number of positive cores, total number of cores and stage).

Main outcome measurement will be number of upgrading, i.e. number of detected ISUP ≥ 2 cancers.

4.1 Study size

The total study size is 400 men recruited from clinics in Norway, Denmark, Finland, and Sweden.

4.2 Participation criteria

4.2.1 Inclusion criteria

- Men diagnosed with low-risk PC and currently on AS and planned for follow-up with systematic and/or fusion biopsies as following standard of care.

4.2.2 Exclusion criteria

- History of treatment for prostate cancer; surgery, radiation, chemotherapy or hormonal treatment.
- Severe illnesses such as metastatic cancers, severe cardio-vascular disease or dementia

5 Statistical analysis plan

5.1 Objective

Demonstrate that the Stockholm3 test can detect ISUP upgrading in an AS cohort with the ultimate aim of demonstrating the safety of a minimally invasive AS protocol where men are biopsied only if they have a positive Stockholm3 test.

5.2 Analysis

We will follow the analysis plan specified below:

5.2.1 Show association for Stockholm3 and upgrading

Test for association between the Stockholm3 score and upgrading using logistic regression

5.2.2 Compare association between Stockholm3 and PSA for upgrading

Compare the strength of the association between the Stockholm3 score and upgrading with the association between the PSA and upgrading using logistic regression with Stockholm3 and PSA as predictors, both standardized to have unit variance. We will test for significant differences in the absolute size of the regression coefficients. Moreover, we will compare the area under the respective receiver operating characteristics curves.

5.2.3 Show sensitivity versus specificity for Stockholm3 and upgrading

We will compute the sensitivity and specificity for detecting upgrading using different Stockholm3 cutoffs for biopsy referrals.

We will stratify all analyses based on MRI conducted or not.

5.3 Power

We used data from the STHLM3 trial to simulate data that we expect to see in STHLM3-AS NorDCaP. Assuming 20% upgrading in STHLM3-AS NorDCaP, we repeatedly sampled dataset with 400 men from the STHLM3 study where 80% of the men had an ISUP 1 cancer and 20% had an ISUP ≥ 2 cancer (representing the upgraded men). In STHLM3 we have the Stockholm3 score for all men and could therefore test for the association between the Stockholm3 score and upgrading. Based on 1,000 resampled datasets, the power for detecting a significant association between Stockhom3 and upgrading is more than 90% with a sample size of 400 men (and an alpha of 0.05). We will also have more than 80% power to test for differences in the strength of the association for the Stockholm3 score and PSA.

6 Study process

6.1 Step 1: Selection and Invitation

Men diagnosed with low-risk PC, currently on AS and planned for follow-up with biopsies will be invited to the study. Potential participants will be invited to the study by their urologist at their yearly follow up. The patient will receive oral and written information about the study. The

written information includes:

1. Invitation letter with brief information about the study
2. Study information brochure with extensive information on the study
3. Consent form

The research participant signs the consent form.

6.2 Step 2: Blood Sampling

At the time when blood for PSA test is drawn, the patient will leave an additional blood test for the Stockholm3-test. The Stockholm3 blood test is sent to A23 Lab in Sweden for analysis.

6.3 STEP 3: Follow-up

Participants will undergo follow-up according to standard of care for AS.

6.4 Step 4: Data collection

The following data will be collected:

- Stockholm3 Risk Score
- Stockholm3 Prostate volume cut-off
- Total PSA value
- If conducted, results from MRI (PI-RADS)
- Results from biopsy, i.e. ISUP, prostate volume, cancer length, number of positive cores, total number of cores, and stage

Data will be collected on a paper CRF

7 Ethical considerations

7.1 Is there an increased risk of missing aggressive cancers?

Patients will undergo surveillance according to standard of care, thus no risk of missing aggressive cancers is present.

7.2 Is there a risk of over-treatment?

Potential participants will be invited to the study by their urologist at their yearly follow up. Thus, no extra biopsy will be undertaken.

7.3 How do we protect the personal integrity?

Information obtained in the study will be collected in one database. The purpose of the database is to collect study data in a proper and safe way for a long time. All information about the participants will be treated with utmost confidentiality and with strong safeguards to preserve their anonymity. Information that can be used to identify the participant (such as name, address and social security numbers) is always kept separate from other data (such as survey responses and blood tests). All data and test results will be treated to prevent unauthorized access to them. The samples will get a unique code so that outsiders cannot identify them. The participants' samples are treated in accordance with the National Biobank Act and other relevant laws.

Everyone who works with STHLM3 AS NorDCaP are under confidentiality agreements. Results from the study are presented only as statistics in which individual answers cannot be traced.

Treatment of the personal data is in accordance with GDPR and other relevant national laws.

8 Timeline

- 1Q2019 Ethics application
- 1Q2019 Start of inclusion
- 4Q2019 End of inclusion
- 1Q2019 Submission of publication

9 References

1. Ferlay J, Soerjomataram I, Dikshit R, et al. Cancer incidence and mortality worldwide: Sources, methods and major patterns in GLOBOCAN 2012. Int J Cancer 2014;:n/a–n/a.
2. Mistry K, Cable G. Meta-analysis of prostate-specific antigen and digital rectal examination as screening tests for prostate carcinoma. J Am Board Fam Pract 2003;16(2):95–101.
3. Schroder FH, Hugosson J, Roobol MJ, Tammela TL, Zappa M, Nelen V, et al. Screening and prostate cancer mortality: results of the European Randomised Study of Screening for Prostate Cancer (ERSPC) at 13 years of follow-up. Lancet. 2014.
4. Hugosson J, Carlsson S, Aus G, et al. Mortality results from the Göteborg randomised population-based prostate-cancer screening trial. Lancet Oncol 2010;11(8):725–32.
5. Nationell kvalitetsrapport för diagnosår 2012 [Internet]. Regionala Cancercentrum i Samverkan; 2013. Available from: http://npcr.se/wp-content/uploads/2013/04/20131121-NPCR-Rapport-2012.pdf
6. Grönberg H, Adolfsson J, Aly M, et al. Prostate cancer screening in men aged 50-69 years (STHLM3): a prospective population-based diagnostic study. Lancet Oncol 2015;16(16):1667–76.
7. Rider JR, Sandin F, Andrén O, Wiklund P, Hugosson J, Stattin P. Long-term outcomes among noncuratively treated men according to prostate cancer risk category in a nationwide, population-based study. Eur Urol [Internet] 2013;63(1):88–96. Available from:http://www.sciencedirect.com.proxy.kib.ki.se/science/article/pii/S0302283812009256/pdfft?md5=babc39ae400d067768cc13a694e3925b&pid=1-s2.0-S0302283812009256-main.pdf
8. Loeb S, Bjurlin MA, Nicholson J, et al. Overdiagnosis and overtreatment of prostate cancer. Eur Urol 2014;65(6):1046–55.
9. Soares R, Di Benedetto A, Dovey Z, Bott S, McGregor RG, Eden CG. Minimum 5-year follow-up of 1138 consecutive laparoscopic radical prostatectomies. BJU Int 2015;115(4):546–53.
10. Aly M, Dyrdak R, Nordström T, et al. Rapid increase in multidrug-resistant enteric bacilli blood stream infection after prostate biopsy-A 10-year population-based cohort study. Prostate 2015;75(9):947–56.
11. Johansson JE, Andren O, Andersson SO, Dickman PW, Holmberg L, Magnuson A, et al. Natural history of early, localized prostate cancer. JAMA. 2004;291(22):2713-9.
12. Kessler B, Albertsen P. The natural history of prostate cancer. Urol Clin North Am. 2003;30(2):219-26.
13. Draisma G, Boer R, Otto SJ, van der Cruijsen IW, Damhuis RA, Schroder FH, et al. Lead times and overdetection due to prostate-specific antigen screening: estimates from the European Randomized Study of Screening for Prostate Cancer. J Natl Cancer Inst. 2003;95(12):868-78.
14. Wilt TJ, Brawer MK, Jones KM, Barry MJ, Aronson WJ, Fox S, et al. Radical prostatectomy versus observation for localized prostate cancer. The New England journal of medicine. 2012;367(3):203-13.
15. Ross HM, Kryvenko ON, Cowan JE, Simko JP, Wheeler TM, Epstein JI. Do adenocarcinomas of the prostate with Gleason score (GS) </=6 have the potential to metastasize to lymph nodes? Am J Surg Pathol. 2012;36(9):1346-52.
16. Klotz L. Active surveillance for prostate cancer: overview and update. Curr Treat Options Oncol. 2013;14(1):97-108.
17. Ellis CL, Walsh PC, Partin AW, Epstein JI. Multiple cores of Gleason score 6 correlate with favourable findings at radical prostatectomy. BJU international. 2013;111(8):E306-9.
18. Prostate cancer National care program of Sweden. http://www.cancercentrum.se. Regional Cancer Centers of Sweden in collaboration. 2014.

19 Berg KD, Toft BG, Roder MA, Brasso K, Vainer B, Iversen P. Prostate needle biopsies: interobserver variation and clinical consequences of histopathological re-evaluation. Apmis. 2011;119(4-5):239-46.

20. Shapiro RH, Johnstone PA. Risk of Gleason grade inaccuracies in prostate cancer patients eligible for

active surveillance. Urology. 2012;80(3):661-6.

21. Duffield AS, Lee TK, Miyamoto H, Carter HB, Epstein JI. Radical prostatectomy findings in patients in whom active surveillance of prostate cancer fails. The Journal of urology. 2009;182(5):2274-8.

22. Anderson J, Burney S, Brooker JE, Ricciardelli LA, Fletcher JM, Satasivam P, et al. Anxiety in the management of localised prostate cancer by active surveillance. BJU international. 2014;114 Suppl 1:55-61.

23. van den Bergh RC, Essink-Bot ML, Roobol MJ, Wolters T, Schroder FH, Bangma CH, et al. Anxiety and distress during active surveillance for early prostate cancer. Cancer. 2009;115(17):3868-78.
