# Supplementary material for: STHLM3 NorDCaP: prospective multicentre trial of Stockholm3 for active surveillance biopsy guidance
Source: BJU Int. 2026 Jul 1;138(3):471–9. doi: 10.1111/bju.70366 (PMC13458658; doi:10.1111/bju.70366)
Supplement: Supplementary file 2 [file BJU-138-471-s001.docx]

**Supplementary Appendix**

**STHLM3 NorDCaP: Prospective Multicenter Trial of Stockholm3 for Active Surveillance Biopsy Guidance**

**Supplementary Figure 1:** Study design

**Supplementary Figure 2:** Boxplot of Stockholm3 and PSA for the active surveillance study cohort stratified by upgrade on biopsy.

**Supplementary Table 1. Contingency tables and diagnostic performance of Stockholm3 and comparator markers for prediction of upgrading to Grade Group ≥2**

|  |  | **Disease  ISUP GG ≥2** | |  | **Statistic** | | **95% CI** |
| --- | --- | --- | --- | --- | --- | --- | --- |
| **Stockholm3 ≥ 11** | Test | Disease + | Disease - | Total | Sn | 71/72 = 0.99 | 0.93, 1.00 |
|  | + | 71 | 113 (FP) | 184 | Sp | 14/127 = 0.11 | 0.06, 0.18 |
|  | - | 1 (FN) | 14 | 15 | PPV | 71/184 = 0.39 | 0.32, 0.46 |
|  |  | 72 | 127 | 199 | NPV | 14/15 = 0.93 | 0.68, 1.00 |
| **Stockholm3 ≥ 15** | Test | Disease + | Disease - | Total | Sn | 67/72 = 0.93 | 0.85, 0.98 |
|  | + | 67 | 94 (FP) | 161 | Sp | 33/127 = 0.26 | 0.19, 0.35 |
|  | - | 5 (FN) | 33 | 38 | PPV | 67/161 = 0.42 | 0.34, 0.50 |
|  |  | 72 | 127 | 199 | NPV | 33/38 = 0.87 | 0.72, 0.96 |
| **PSA Density ≥ 0.10** | Test | Disease + | Disease - | Total | Sn | 53/70 = 0.76 | 0.64, 0.85 |
|  | + | 53 | 63 (FP) | 116 | Sp | 55/118 = 0.47 | 0.37, 0.56 |
|  | - | 17 (FN) | 55 | 72 | PPV | 53/116 = 0.46 | 0.36, 0.55 |
|  |  | 70 | 118 | 188 | NPV | 55/72 = 0.76 | 0.65, 0.86 |
| **PSA Density ≥ 0.15** | Test | Disease + | Disease - | Total | Sn | 42/70 = 0.60 | 0.48, 0.72 |
|  | + | 42 | 33 (FP) | 75 | Sp | 85/118 = 0.72 | 0.63, 0.80 |
|  | - | 28 (FN) | 85 | 113 | PPV | 42/75 = 0.56 | 0.44, 0.67 |
|  |  | 70 | 118 | 188 | NPV | 85/113 = 0.75 | 0.66, 0.83 |
| **PSA Doubling Time < 3 years** | Test | Disease + | Disease - | Total | Sn | 38/70 = 0.54 | 0.42, 0.66 |
|  | + | 38 | 79 (FP) | 117 | Sp | 44/123 = 0.36 | 0.27, 0.45 |
|  | - | 32 (FN) | 44 | 76 | PPV | 38/117 = 0.32 | 0.24, 0.42 |
|  |  | 70 | 123 | 193 | NPV | 44/76 = 0.58 | 0.46, 0.69 |

FP = false positive; FN = false negative; Sn = sensitivity; Sp = specificity; PPV = positive predictive value; NPV = negative predictive value; CI = confidence interval; PSA = prostate-specific antigen; ISUP GG = International Society of Urological Pathology Grade Group

**Supplementary Table 2.** Sensitivity, specificity, positive predictive value, negative predictive value, and area under the receiver operating characteristic curve (AUC) of the various tests for predicting prostate cancer upgrading at rebiopsy in high-risk disease (defined as ISUP grade group ≥3 or ISUP grade group 2 with cancer length ≥10 mm and/or >10% Gleason pattern 4).

| Biomarker/Test | Threshold | Sensitivity (95% CI) | Specificity (95% CI) | AUC (95% CI) | PPV (95% CI) | NPV (95% CI) |
| --- | --- | --- | --- | --- | --- | --- |
| Stockholm3 | ≥11 | 1.00 (0.86-1.00) | 0.09 (0.05-0.14) | 0.65 (0.55-0.76) | 0.14 (0.09-0.19) | 1.00 (0.78-1.00) |
|  | ≥15 | 0.96 (0.80-1.00) | 0.21 (0.15-0.28) |  | 0.15 (0.10-0.21) | 0.97 (0.86-1.00) |
| PSA Density | ≥0.10 | 0.83 (0.61-0.95) | 0.41 (0.34-0.49) | 0.72 (0.61-0.83) | 0.16 (0.10-0.24) | 0.94 (0.86-0.98) |
|  | ≥0.15 | 0.74 (0.52-0.90) | 0.65 (0.57-0.72) |  | 0.23 (0.14-0.34) | 0.95 (0.89-0.98) |
| PSA Doubling time | <3 yrs | 0.48 (0.27-0.69) | 0.38 (0.30-0.45) | 0.58 (0.45-0.70) | 0.10 (0.05-0.17) | 0.83 (0.73-0.91) |
| MRI | PI-RADS ≥3 | 1.00 (0.84-1.00) | 0.25 (0.17-0.34) | 0.68 (0.57- 0.79) | 0.18 (0.12-0.27) | 1.00 (0.88-1.00) |
|  | PI-RADS ≥4 | 0.86 (0.64-0.97) | 0.50 (0.41-0.59) |  | 0.23 (0.14-0.34) | 0.95 (0.87-0.99) |
